# Supplementary material for: SequelTools: a suite of tools for working with PacBio Sequel raw sequence data
Source: BMC Bioinformatics. 2020 Oct 1;21:429. doi: 10.1186/s12859-020-03751-8 (PMC7532105; doi:10.1186/s12859-020-03751-8)
Supplement: Supplementary file 1 — Additional file 1. [file 12859_2020_3751_MOESM1_ESM.pdf]

SequelTools Supplementary Materials

David E. Hufnagel, Matthew B. Hufford, and Arun S. Seetharam

July 2020

Table S1: Summary statistics table part 1

SMRTcell	numReadsCLR	numReadsSubedCLR	numReadsSubread
m54138_180610_050652	666453	372032	437596
m54138_180610_152029	748962	454753	544731
m54138_180619_053104	773389	484816	575245
m54138_180620_123320	915891	679218	781352
m54138_180620_225343	904201	628103	723088
m54138_180621_091000	820010	570454	653655
m54138_180627_120814	766783	500556	585498
m54138_180627_222429	769051	469297	556407

Table S2: Summary statistics table part 2

numReadsLongestSub	totalBasesCLR	totalBasesSubedCLR	totalBasesSubread
372032	9903912080	9066577847	6156338170
454753	1.2677E+10	1.1152E+10	6796863421
484816	1.3808E+10	1.2595E+10	7479347974
679218	2.4638E+10	2.1686E+10	8673538367
628103	2.2217E+10	1.9391E+10	8173394973
570454	1.7348E+10	1.5889E+10	8329279410
500556	1.3653E+10	1.2458E+10	7384567281
469297	1.2856E+10	1.1583E+10	6749552695

Table S3: Summary statistics table part 3

totalBasesLongestSub	meanReadLenCLR	meanReadLenSubedCLR
5.878E+09	14861	24370
6.483E+09	16926	24524
7.014E+09	17854	25979
8.239E+09	26901	31928
7.764E+09	24571	30873
7.907E+09	21156	27852
6.96E+09	17806	24887
6.358E+09	16717	24682

Table S4: Summary statistics table part 4

meanReadLenSubread	meanReadLenLongestSub	medianReadLenCLR
14069	15801	9027
12477	14256	13233
13002	14467	13901
11101	12130	24471
11303	12362	21699
12743	13861	18831
12612	13904	13181
12131	13549	11390

Table S5: Summary statistics table part 5

medianReadLenSubedCLR	medianReadLenSubread	medianReadLenLongestSub
22172	11228	14073
22051	9109	11907
23112	10047	12210
29120	7948	9327
27722	8195	9635
25018	9901	11489
22110	9307	11020
21798	8840	10733

Table S6: Summary statistics table part 6

n50CLR	n50SubedCLR	n50Subread	n50LongestSub	l50CLR
31280	31631	23049	23612	110089
31778	32373	21804	22341	138071
33914	34393	21730	22391	141188
41020	41673	19646	20203	218456
39894	40787	19880	20433	199129
36029	36629	21177	21706	171283
33912	34374	21517	22200	140570
33421	34017	21077	21747	132950

Table S7: Summary statistics table part 7

l50SubedCLR	l50Subread	l50LongestSub	PSR	ZOR
100336	93650	87778	0.955	0.85
120233	109438	102443	0.954	0.835
127745	120785	110533	0.938	0.843
190145	153025	142368	0.95	0.869
171306	143674	133758	0.95	0.869
155271	138592	128978	0.949	0.873
127325	119017	109528	0.942	0.855
118603	111464	102569	0.942	0.843

Table S8: Assembly statistics for raw reads and reads filtered by CLR length using the Read Filtering tool

Metrics	All Reads	CLR >= 5Kb
Number of scaffolds	555	659
Total size of scaffolds	124,850,704	126,349,435
Total scaffold length	92.5%	93.6%
(% of 135Mb assumed genome size)		
Longest scaffold	4,243,121	4,243,141
Shortest scaffold	2,357	6,520
Number of scaffolds > 1Kb	555	659
Number of scaffolds > 10Kb	528	649
Number of scaffolds > 100Kb	219	253
Number of scaffolds > 1Mb	30	24
Mean scaffold size	224,956	191,729
Median scaffold size	65,852	62,543
N50 scaffold length	699,106	576,605
L50 scaffold count	46	60

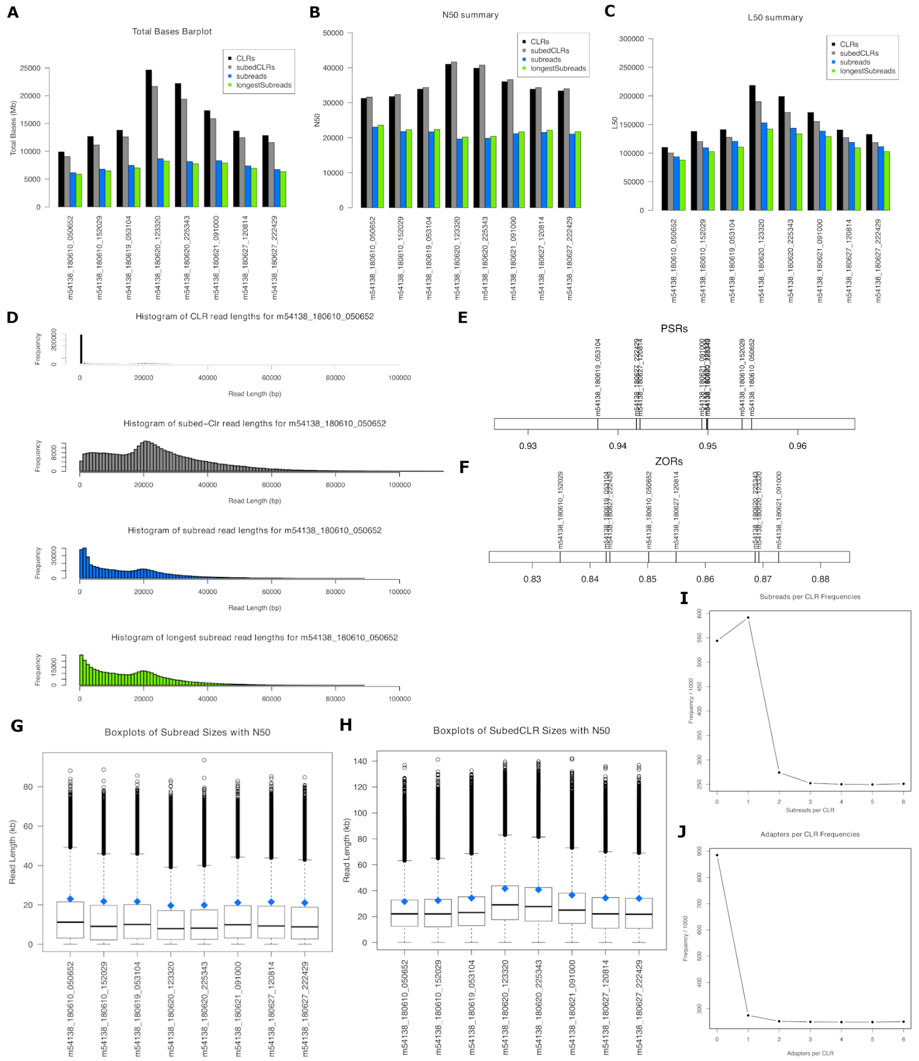

Figure S1: All plots generated by *SequelTools*. (A) A barplot of the sum of read lengths, (B) A barplot of N50s, (C) A barplot of L50s, (D) Read length histograms for m54138_180610_050652, (E) A two dimensional plot of PSRs, (F) A two dimensional plot of ZORs, (G) Boxplots of subread lengths with N50s as blue diamonds, (H) Boxplots of subedCLR lengths with N50s as blue diamonds, (I) A frequency plot showing the distribution of subreads per CLR, and (J) A frequency plot showing the distribution of adapters per CLR, for all SMRTcells in our benchmarking data set.
